# Arabidopsis Ubiquitin-Conjugating Enzymes UBC4, UBC5, and UBC6 Have Major Functions in Sugar Metabolism and Leaf Senescence

**DOI:** 10.3390/ijms231911143

**Published:** 2022-09-22

**Authors:** Sheng Wang, Ling Cao, Ian R. Willick, Hong Wang, Karen K. Tanino

**Affiliations:** 1 Department of Plant Sciences, University of Saskatchewan, Saskatoon, SK S7N 5E5, Canada; 2 Department of Biochemistry, Microbiology and Immunology, University of Saskatchewan, Saskatoon, SK S7N 5E5, Canada

**Keywords:** Arabidopsis, fructose 1,6-bisphosphatase (FBPase), leaf senescence, sugar metabolism, ubiquitin-conjugating enzyme, UBC4, UBC5, UBC6

## Abstract

The ubiquitin-conjugating enzyme (E2) is required for protein ubiquitination. Arabidopsis has 37 E2s grouped into 14 subfamilies and the functions for many of them are unknown. We utilized genetic and biochemical methods to study the roles of Arabidopsis UBC4, UBC5, and UBC6 of the E2 subfamily IV. The Arabidopsis *ubc4/5/6* triple mutant plants had higher levels of glucose, sucrose, and starch than the control plants, as well as a higher protein level of a key gluconeogenic enzyme, cytosolic fructose 1,6-bisphosphatase 1 (cyFBP). In an in vitro assay, the proteasome inhibitor MG132 inhibited the degradation of recombinant cyFBP whereas ATP promoted cyFBP degradation. In the quadruple mutant *ubc4/5/6 cyfbp*, the sugar levels returned to normal, suggesting that the increased sugar levels in the *ubc4/5/6* mutant were due to an increased cyFBPase level. In addition, the *ubc4/5/6* mutant plants showed early leaf senescence at late stages of plant development as well as accelerated leaf senescence using detached leaves. Further, the leaf senescence phenotype remained in the quadruple *ubc4/5/6 cyfbp* mutant. Our results suggest that UBC4/5/6 have two lines of important functions, in sugar metabolism through regulating the cyFBP protein level and in leaf senescence likely through a cyFBP-independent mechanism.

## 1. Introduction

Ubiquitination is a fundamental process responsible for regulating protein turnover and homeostasis in eukaryotes including plants. A single ubiquitin (Ub) or a poly-Ub chain (in polyubiquitination) is attached to a substrate protein. In addition, in polyubiquitination, different Ub-Ub linkages have distinct conformational structures that could be recognized by various Ub-binding proteins, leading to diverse outcomes of the ubiquitinated proteins [1]. Protein ubiquitination requires three types of enzymes biochemically: Ub-activating enzyme (E1), Ub-conjugating enzyme (E2), and Ub ligase (E3). While the E3 ligases are generally believed to determine the substrate specificity, the E2s function with an E3 to transfer a Ub or Ub chain to substrates and to also determine the specificity of the Ub chain linkages [2]. Our understanding of E2 functions in plants is still very limited, but not surprisingly evidence so far suggests that they function in diverse processes in plants [3]. Most of the understanding on plant E2s has been obtained from the studies of Arabidopsis E2s. There are 37 Arabidopsis E2s encoded by the *UBC* genes which can be divided into 14 subgroups, subfamilies III to XVI, except for the subgroups I and II which are categorized for the UBC domain-containing proteins functioning in RUB1 and SUMO conjugation pathways [4,5].

Among the 14 subfamilies of Arabidopsis E2s, functions have been reported for some subfamilies or at least one member of a subfamily. UBC1 and UBC2, belonging to the subfamily III, have been shown to be involved in histone 2B monoubiquitination and flowering time regulation [6,7,8] as well as in the salt stress response by affecting the expression of the stress responsive transcription factors MYB42 and MAPK4 (MITOGEN-ACTIVATED PROTEIN KINASE) [9]. UBC7, UBC13, and UBC14 in the subfamily V are critical for stress responses and the triple mutant plants were found to be hypersensitive to several environmental stress conditions and abscisic acid (ABA) [10]. The sensitivity of the mutant plants to the endoplasmic reticulum (ER) stress inducers such as tunicamycin and the sequence similarity with yeast Ubc7 and the homologs in animals which are a core component in the ERAD (ER-associated degradation) pathway suggest that UBC7/13/14 function in the plant ERAD pathway [10]. In yeast, two E2s are involved in ERAD: Ubc7 works with the E3 Hrd1 complex to remove substrates in the lumen and membrane, whereas E3 Doa10 together with both Ubc6 and Ubc7 removes substrates in the cytosolic domain of the ER [11,12]. UBC32, one of the three members in the subfamily XIV, was identified as a plant homolog of yeast Ubc6 and is involved in the polyubiquitination and degradation of ERAD substrates [13]. In addition, UBC32 can modulate the level of AtOS9, a component of the Arabidopsis HRD1 complex, for ERAD tuning [14]. Information for the other two members, UBC33 and UBC34, is very limited, but UBC34 was recently reported to regulate the turnover of SUC2 (SUCROSE TRANSPORTER 2) and thus affect sucrose transport [15].

The subfamily IX only has one member UBC21 which was initially identified as PEX4 (PEROXIN 4) [16] and recent evidence indicates that UBC21 functions in the ubiquitination and retrotranslocation of the peroxisome matrix protein receptor, PEX5 [17]. As the sole member of subfamily X, UBC22 shares a significant sequence similarity with the animal and human E2s, which can catalyze Lys11-linked polyubiquitination. Indeed, UBC22 could catalyze Lys11-linked Ub dimer formation in vitro, suggesting it as a unique plant E2 responsible for Lys11-linked ubiquitination [18]. Studies of *ubc22* mutants have revealed important roles of UBC22 in female gametophyte development and meiosis as well as in plant immunity [18,19,20]. UBC24, one of the four members in the subfamily XI (UBC23 to 26), was originally identified as PHO2 (PHOSPHATE OVERACCUMULATOR 2) to ubiquitinate and degrade the inorganic phosphate (Pi) transporter PT2 [21,22]. Recently, ORE1 (ORESARA 1) with an important role in leaf senescence during nitrogen deficiency was identified as another target of UBC24 [23]. UBC26 from the subfamily XI and UBC27, the only member of the subfamily XII, were recently demonstrated to be involved in ABA signaling [24,25]. UBC35 and UBC36, the two members of the subfamily XV, are closely related to the UBC13 proteins of non-plant species and thus are also referred to as UBC13A and UBC13B [26]. These two E2s are involved in Lys63-linked ubiquitination in Arabidopsis [26,27] and have multiple functions in plant growth and stress responses [27,28,29,30,31].

Still, the specific cellular and biological functions for many E2s remain unknown. UBC4, UBC5, and UBC6 (UBC4/5/6 for simplicity) are the three members of the subfamily IV. Their gene expression pattern was initially analyzed more than two decades ago [32,33]. However, no information is available on their biological functions. Based on the protein sequences, UBC4/5/6 fall into the same phylogenetic group with the yeast E2 Ubc8 [4], which plays an important role in the glucose-induced degradation of a key gluconeogenic enzyme, fructose 1,6-bisphosphatase 1 (Fbp1) [34,35].

In C3 plants, carbon dioxide is fixed in the chloroplast during the Calvin–Benson Cycle, which ultimately leads to the production of triose phosphates. A fraction of these triose phosphates is catabolized for ribulose-1,5-bisphosphate regeneration and the remainder is stored as starch inside the chloroplast or is exported to the cytosol for sucrose synthesis [36]. Some Calvin–Benson Cycle enzymes, including the fructose-1,6-bisphosphatase (FBPase), are the key regulators to maintain a balance between the export and regeneration of ribulose-1,5-bisphosphates [37]. In brief, the FBPase catalyzes the conversion of fructose 1,6-bisphosphate (F1,6BP) to fructose-6-phosphate (F6P) with the release of a Pi. Two types of FBPases exist in plants. The chloroplastic FBPase plays a key role in the Calvin–Benson pathway for the generation of ribulose-1,5-bisphosphate and formation of F6P in starch synthesis, while the cytosolic FBPase is involved in catalyzing the first irreversible reaction in sucrose synthesis from triose phosphates [38]. In Arabidopsis, one cytosolic FBPase gene (*cyFBP*) and two chloroplastic FBPase genes (*cFBP1* and *cFBP2*) have been identified [39]. *cFBP2* is similar to *cFBP1* but with a much lower expression level, suggesting that *cFBP1* plays a major role in Arabidopsis [39]. In *cyfbp* and *cfbp1* single and *cyfbp cfbp1* double mutants, the F1,6BP levels were higher compared to the wild-type (WT) plants since the conversion of F1,6BP to F6P was affected in these mutants [39]. Conversely, the levels of soluble sugars (glucose, fructose, and sucrose) were lower in those mutants. The reported growth phenotypes of these mutants were different in that the *cyfbp* mutant was similar to the WT while the *cfbp1* mutant and *cyfbp cfbp1* double mutant showed a dwarf phenotype [39].

Due to the important functions of these two types of FBPase in plants, there have been studies to investigate the effects of overexpressing an FBPase either in the cytosol or chloroplast. The transgenic tobacco plants expressing a cyanobacterial FBPase-II in the chloroplast displayed enhanced photosynthetic activities and an increased starch level in leaves [40]. Additionally, overexpressing a cyanobacterial fructose-1,6-/sedoheptulose-1,7-bisphosphatase in tobacco resulted in increased FBPase activity in the cytosol and enhanced sucrose synthesis at elevated CO_2_ concentrations [41]. In addition, the transgenic Arabidopsis plants overexpressing both *TPT (TRIOSE PHOSPHATE/PHOSPHATE TRANSLOCATOR)* and *cyFBP* exhibited enhanced growth and increased levels of soluble sugars [42]. These results suggest that the regulation and levels of FBPases are important for sugar metabolism and photosynthesis in plants.

The transcript level of cytosolic FBPase in plants is regulated developmentally and environmentally [43] as well as through the end-product repression [44]. The FBPase may also be regulated post-translationally. Phosphorylation and dephosphorylation are known to be an important means for regulating the activity of FBPases in yeast and animals [45,46]. However, experimental evidence for the phosphorylation and dephosphorylation regulation of plant FBPases is lacking. On the other hand, the activity of chloroplastic FBPase may be regulated by the formation or removal of disulfide bonds through the interaction with thioredoxin [47,48].

In this study, we investigated the functions of Arabidopsis UBC4/5/6. We present experimental results demonstrating that these E2s have important functions in sugar metabolism and also leaf senescence. Furthermore, we showed that they are involved in regulating the protein level of cyFBP, but interestingly not that of cFBP1, thus unraveling a mechanism through which UBC4/5/6 affect sugar levels in plants.

## 2. Results

### 2.1. Characterization of UBC4/5/6 and Their T-DNA mutants

Arabidopsis UBC4, UBC5, and UBC6 (encoded by *At5g41340*, *At1g63800*, and *At2g46030,* respectively) [4] are closely related to each other and have similar sizes (Figures S1 and S2). The three E2s also share high levels of sequence similarity with the human E2 UBE2H and yeast E2 Ubc8, particularly in their UBC domain (Figure S1), suggesting that these E2s from different species are homologs. It is interesting to note that the C-terminal sequences of UBE2H and Ubc8 vary considerably from those of the Arabidopsis E2s. Due to the extra sequence in the C-terminus, the yeast Ubc8 is the largest protein. In addition, phylogenetic analysis of the three Arabidopsis E2s revealed that UBC4 and UBC5 were more closely related to each other than to UBC6 (Figure S2).

**Figure 1:**
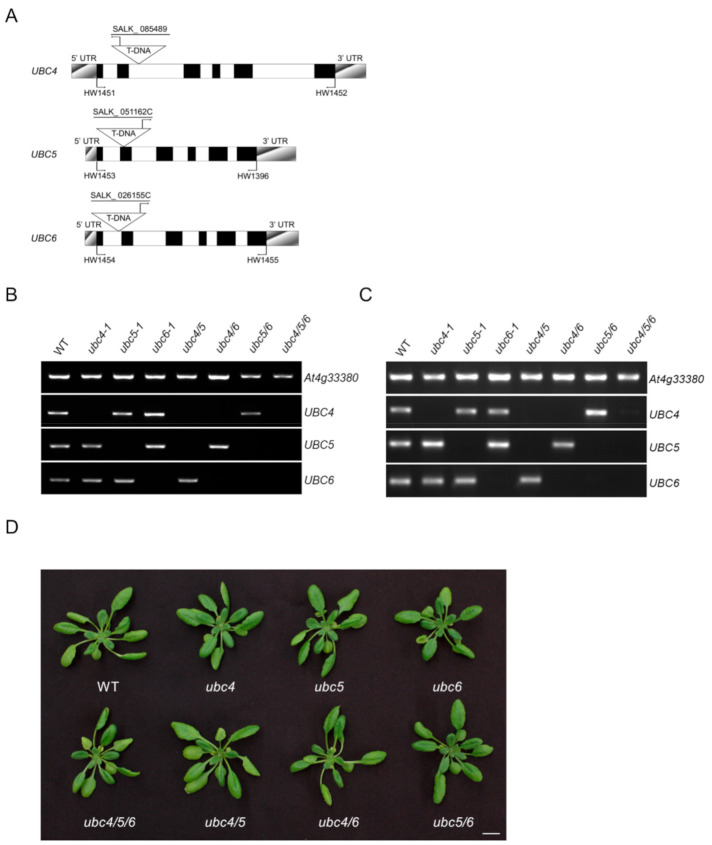
Characterization of *ubc4, ubc5, and ubc6* mutants. (**A**) Schematic representation of the genomic structures of *UBC4*, *UBC5*, and *UBC6* with the locations of primers and insertion sites of T-DNA in *ubc4-1*, *ubc5-1*, and *ubc6-1* mutants. Primers 1451/1452, 1453/1396, and 1454/1455 are the gene specific primers for *UBC4*, *UBC5*, and *UBC6*, respectively. Closed boxes, exons; open boxes, introns; shadowed boxes, untranslated regions. (**B**) Analysis of *ubc4-1*, *ubc5-1*, *ubc6-1*, *ubc4/5*, *ubc4/6, ubc5/6*, and *ubc4/5/6* mutant lines by genomic PCR. Gene-specific primers were used and PCR products were run in 1% agarose gels. The mutant lines used are indicated above the panels and specific genes analyzed are indicated at the right of the panels. The gene *At4g33380* was used as a control. (**C**) Analysis of *ubc4-1*, *ubc5-1*, *ubc6-1, ubc4/5*, *ubc4/6*, *ubc5/6*, and *ubc4/5/6* mutant lines by RT-PCR. cDNAs were prepared from total RNA samples and gene-specific primers were used for amplifying the cDNA of the perspective WT gene. The transcript level of *At4g33380*, as shown in the upper panel, was used as a reference. (**D**) Representative images showing plants of *ubc4-1*, *ubc5-1*, *ubc6-1*, *ubc4/5*, *ubc4/6*, *ubc5/6*, and *ubc4/5/6* mutants. Pictures were taken at the 30-day stage. Scale bar = 1 cm.

To understand the biological function of *UBC4/5/6*, T-DNA insertion mutants for these three genes were obtained from the Arabidopsis Biological Resource Center and genotyped to acquire the homozygous lines (Figure 1A). Genomic DNA PCR and reverse transcription-PCR (RT-PCR) using gene-specific primers of *UBC4*, *UBC5*, and *UBC6* did not amplify the full-length genomic DNA and cDNA sequences of each gene (Figure 1B,C), indicating that each respective gene was disrupted in these mutants by the T-DNA insertion. These mutants are designated *ubc4-1* (for SALK_085489), *ubc5-1* (for SALK_051162C), and *ubc6-1* (for SALK_026155C), respectively. Since UBC4/5/6 are closely related members in the E2 subfamily IV, functional redundancy is likely. Thus, these single mutants were crossed, and different double mutants (*ubc4-1*, *ubc5-1*, *ubc4-1*, *ubc6-1*, and *ubc5-1*, *ubc6-1*) and furthermore the triple mutant (*ubc4-1*, *ubc5-1*, *ubc6-1*) were obtained. For simplicity, these mutants are referred to as *ubc4/5*, *ubc4/6*, *ubc5/6*, and *ubc4/5/6* hereafter. The mutant plants were morphologically similar to the WT plants under normal growth conditions, although they appeared slightly smaller than the WT plants (Figure 1D and Figure S3).

### 2.2. UBC4/5/6 Are Involved in Sugar Metabolism

**Figure 2:**
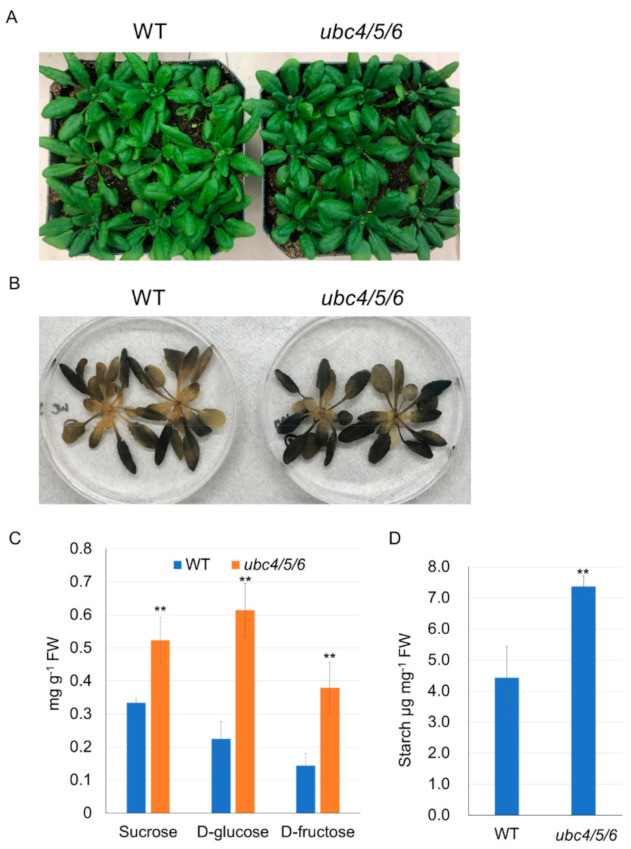
Plant morphology, starch and sugar analyses of the WT and *ubc4/5/6* triple mutant. (**A**) Image to show the WT and *ubc4/5/6* mutant plants at the 5-week stage. (**B**) Starch iodine staining of the WT and *ubc4/5/6* mutant plants. Five-week-old plants were harvested during the day, stained with Lugol solution for 2 min, and cleared for observation. (**C**) Quantitative analyses of sucrose, glucose, and fructose levels in the WT and the *ubc4/5/6* mutant. About 100 mg of leaf tissues from 3-week-old plants were used for extracting soluble sugars. Each datum (in mg of sucrose, glucose, or fructose per gram of fresh tissues) represents the average of four biological replicates (error bar = standard deviation). Student’s *t*-test was performed to determine whether there is a significant difference (**: *p* < 0.01). (**D**) Quantification of the starch level in the WT and *ubc4/5/6* mutant. About 100 mg of leaf tissues from 3-week-old plants were used for the starch analysis. Each datum (in mg of starch per gram of fresh tissues) represents the average of four biological replicates (error bar = standard deviation). Student’s *t*-test was performed to determine whether there is a significant difference between the WT and mutant (**: *p* < 0.01).

Interestingly, the *ubc4/5/6* triple mutant plants appeared slightly darker at the 4- to 5-week stage of growth before bolting (Figure 2A). We asked whether the sugar metabolism was affected in the mutant plants, since yeast Ubc8, the putative homolog of UBC4/5/6, is involved in carbohydrate metabolism by regulating the gluconeogenic enzyme Fbp1 [34]. The starch level in the *ubc4/5/6* triple mutant was determined first using an iodine staining method. There was darker iodine staining in the leaves of the triple mutant plants compared to the WT plants, indicating that the mutant leaves had more starch (Figure 2B). We further quantified the soluble sugar and starch levels. As showed in Figure 2C, indeed the *ubc4/5/6* mutant plants at the 3-week stage had higher levels of glucose, fructose, and sucrose than the WT plants. Consistent with the iodine staining result, the triple mutant also had a higher starch level (Figure 2D). To confirm that those changes in soluble sugar and starch levels were due to the inactivation of *UBC4/5/6* genes, *pUBC4::UBC4-GUS* (*UBC4-GUS* fusion used for easy detection) was introduced into the *ubc4/5/6* mutant. The leaf color of the triple mutant plants expressing *pUBC4::UBC4-GUS* was similar to that of the WT plants and the glucose level was completely normal (Figure S4), indicating that those mutant phenotypes were rescued in the mutant plants by expressing *pUBC4::UBC4-GUS*. Collectively, these results suggest that UBC4/5/6 have an important role in sugar metabolism in plants. Additionally, since cold tolerance is correlated with the soluble sugar levels in plants [49], we determined the tolerance of the triple mutant plants to the freezing temperature stress. Interestingly, more *ubc4/5/6* mutant plants survived after the plants were cooled to and treated at −10 °C for 1 h compared with the WT, suggesting the enhanced freezing tolerance of the triple mutant (Figure S5).

### 2.3. UBC4/5/6 have an Important Role in the Turnover of the Cytosolic FBPase

**Figure 3:**
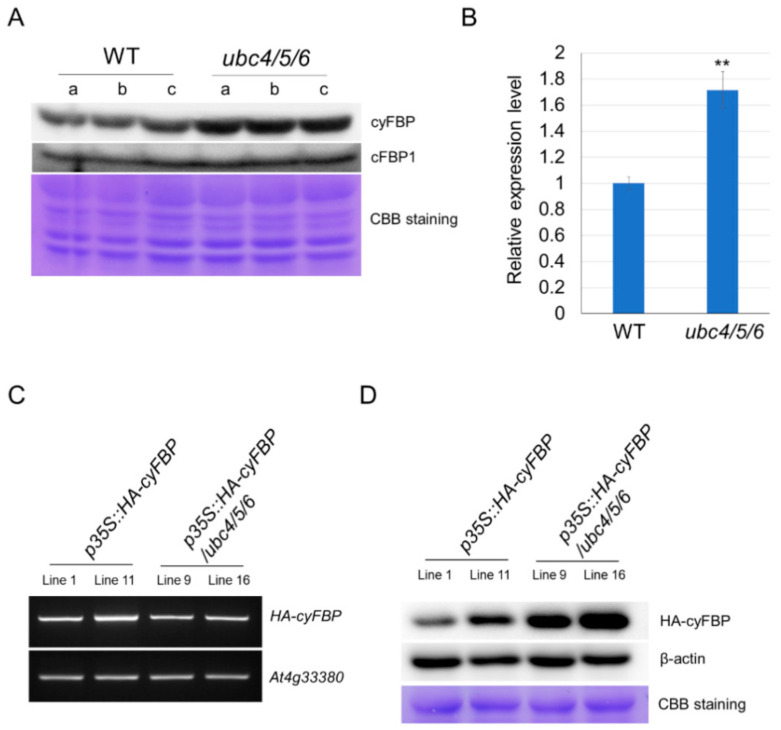
Analysis of cyFBP and cFBP1 in the WT and *ubc4/5/6* mutant. (**A**) Analysis of cyFBP and cFBP1 in the WT and *ubc4/5/6* mutant plants. Total proteins were extracted from mature leaves of the 5-week-old plants and used for Western blotting analysis with anti-cyFBP and anti-cFBP1 antibodies. Three replicate samples from each background were analyzed. The Coomassie Brilliant Blue (CBB) staining of the gel at the lower panel shows the evenness of sample loading. (**B**) Quantitative analysis of the band intensity in (**A**). The intensities of the cyFBP bands were measured using ImageJ. The relative expression level is used for the cyFBP level in the mutant with the level in the WT set as 1.0. The error bars indicate standard deviations. Student’s *t*-test was performed to determine the significance of the difference between the WT and *ubc4/5/6* mutant (**: *p* < 0.01). (**C**) Transcript analysis of *HA-cyFBP* in the WT and *ubc4/5/6* backgrounds. The construct *p35S::HA-cyFBP* was introduced into the WT and *ubc4/5/6* mutant. Transgenic lines (T3) were screened for *HA-cyFBP* expression and two transgenic lines for each background with similar levels of *HA-cyFBP* as determined by RT-PCR were selected. The transcript level of *At4g33380* was used as a reference. (**D**) Analysis of HA-cyFBP protein level in the WT and *ubc4/5/6* backgrounds. Transgenic lines (two for each background) with a similar level of *HA-cyFBP* transcripts as shown in (**C**) were used. Total proteins were extracted from 2-week-old seedlings and the HA-cyFBP protein was detected using Western blotting with an anti-HA antibody (top panel). β-actin protein was used as a loading control (middle panel). Additionally, the gel was stained with Coomassie Brilliant Blue (CBB) to show the evenness of sample loading.

In yeast, the E2 Ubc8 functions in the proteasome degradation of a key gluconeogenic enzyme FBPase [34]. The phylogenetic closeness of UBC4/5/6 to the yeast Ubc8 (Figure S2) led us to ask whether the Arabidopsis E2s may also play a role in regulating the level of plant FBPase. Therefore, we analyzed the protein level of FBPases in the *ubc4/5/6* triple mutant. In Arabidopsis, there is one cytoplasmic FBPase (cyFBP) and two chloroplastic FBPases (cFBP1 and cFBP2) [39]. Since it has been reported that the expression level of *cFBP2* is very low, we analyzed the protein levels of cyFBP and cFBP1 in the *ubc4/5/6* mutant using Western blotting. The antibodies against cyFBP and cFBP1 (from Agrisera) were tested first and were shown to be specific using *cyfbp* and *cfbp1-2* single mutants along with the WT plants (Figure S6). The Western analysis of WT plants showed that cyFBP and cFBP1 levels in the leaves gradually increased as the plants aged and were highest in the leaves and stems of 7-week WT plants (Figure S7). We then compared their levels in mature leaves of 5-week-old WT and *ubc4/5/6* mutant plants. Interestingly, the cyFBP level was significantly higher in the triple mutant plants, whereas the cFBP1 level in the triple mutant was similar to that in the WT (Figure 3A,B). To further confirm this observation, we introduced cyFBP tagged with the hemagglutinin (HA) (in the construct *p35S::HA-cyFBP*) into both the WT and triple mutant. Individual transgenic lines were screened using RT-PCR and lines with similar *HA-cyFBP* transcript levels in the WT and triple mutant background were identified (Figure 3C). The Western blotting results showed that HA-cyFBP protein levels in two transgenic lines in the mutant background were clearly higher than those in the WT background (Figure 3D). Those two independent lines of results consistently showed that the cyFBP protein level increased when *UBC4/5/6* were inactivated in the *ubc4/5/6* mutant plants, suggesting that UBC4/5/6 function in regulating the level of cyFBP in plants.

**Figure 4:**
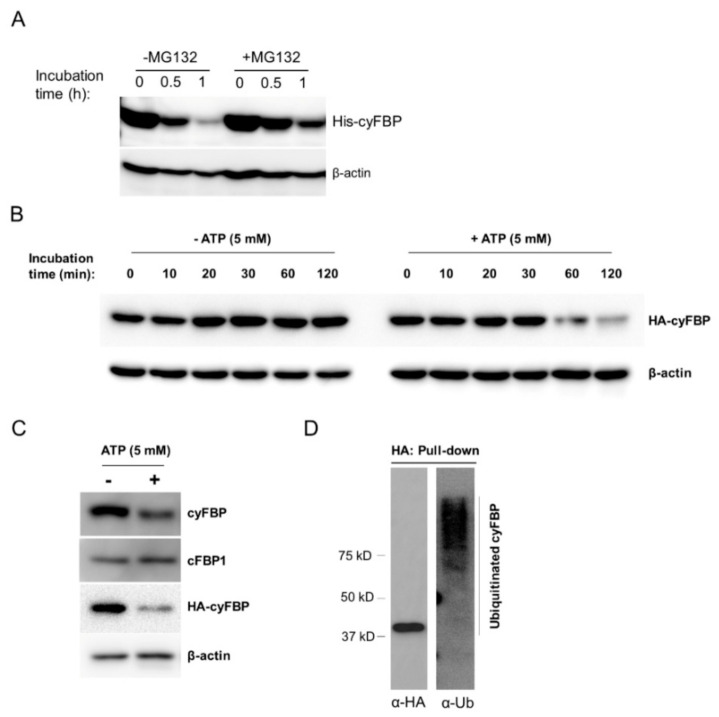
Analysis of cyFBP protein degradation. (**A**) MG132 inhibits the degradation of His-cyFBP in the plant protein extract. The total protein extract was prepared from mature leaf tissues of 6-week-old WT plants. Recombinant His-cyFBP protein (300 ng) was added to the total protein extract (500 μg) in 100 μL of reaction volume with or without MG132 (100 μM). His-cyFBP protein was detected by Western blotting using an anti-His-tag antibody. β-actin was used as a loading control for the total protein extract added. (**B**) Effect of ATP on HA-cyFBP degradation by an in vitro assay. The total protein extract was prepared from the transgenic *p35S::HA-cyFBP* plants. About 60 μg of total proteins in 50 μL of reaction volume were incubated at room temperature (21 °C) with or without (5 mM) ATP for the indicated lengths of time (min). The samples were added with 4X SDS loading buffer to stop the reaction and then used in Western blotting with an anti-HA antibody to detect the HA-cyFBP protein. β-actin protein was detected as a loading control. (**C**) Effect of ATP on the degradation of cyFBP and cFBP1. Total proteins from transgenic *p35S::HA-cyFBP* plants (T3 line) were isolated and incubated with or without (5 mM) ATP for 2 h. cyFBP and cFBP1 proteins were detected using Western blotting with anti-cyFBP and anti-cFBP1 antibodies. The HA-cyFBP was also analyzed as a positive control. β-actin protein was detected as a loading control. (**D**) Detection of HA-cyFBP ubiquitination. Total proteins were extracted from 2-week-old seedlings. The HA-cyFBP protein was pulled down from the total proteins (1 mg) using HA-agarose beads and analyzed by western blotting. The left panel shows the immunoblot with an anti-HA antibody and the right panel shows the blot with an anti-Ub antibody.

To demonstrate whether the degradation of cyFBP is through the 26S proteasome, purified His-tagged cyFBP (His-cyFBP) was incubated with the protein extract from the WT plants in a cell-free degradation assay. The results showed that the His-cyFBP level decreased with incubation time and MG132, an inhibitor of the 26S proteasome, attenuated the degradation of His-cyFBP (Figure 4A). Since ATP is required for protein ubiquitination and adding ATP to the cell extract can enhance protein degradation in a cell-free protein degradation assay [25,50], we performed the protein degradation assay with or without 5 mM ATP. There was increased HA-cyFBP degradation after one hour of incubation in the presence of 5 mM ATP compared to the control treatment without ATP (Figure 4B), suggesting that the added ATP increased the degradation of HA-cyFBP. Furthermore, we also analyzed the endogenous cyFBP and cFBP1 levels in the protein extracts using the cell-free protein degradation assay. Consistent with what was observed for the HA-cyFBP, the endogenous cyFBP level also decreased after one hour of incubation in the presence of 5 mM ATP, while intriguingly the endogenous cFBP1 level was not affected by the addition of ATP (Figure 4C). These results clearly showed that cyFBP protein was degraded in the protein extract, MG132 inhibited the degradation and ATP promoted the degradation, indicating that cyFBP is subjected to 26S proteasome-mediated degradation.

To determine whether cyFBP is ubiquitinated in plant cells, HA-cyFBP protein was pulled down from the total protein extract of the transgenic line expressing *p35S::HA-cyFBP* using HA-conjugated agarose beads. The purified HA-cyFBP protein was analyzed by Western blotting using anti-HA and anti-Ub antibodies. As illustrated in Figure 4D, the Western blot using an anti-HA antibody showed a clear band of the HA-cyFBP and the blot using the anti-Ub antibody showed smearing and ladder-like bands above the HA-cyFBP band, suggesting that the HA-cyFBP is ubiquitinated in vivo.

### 2.4. UBC4/5/6 Function in Sugar Metabolism Is Likely through the Cytosolic FBPase

**Figure 5:**
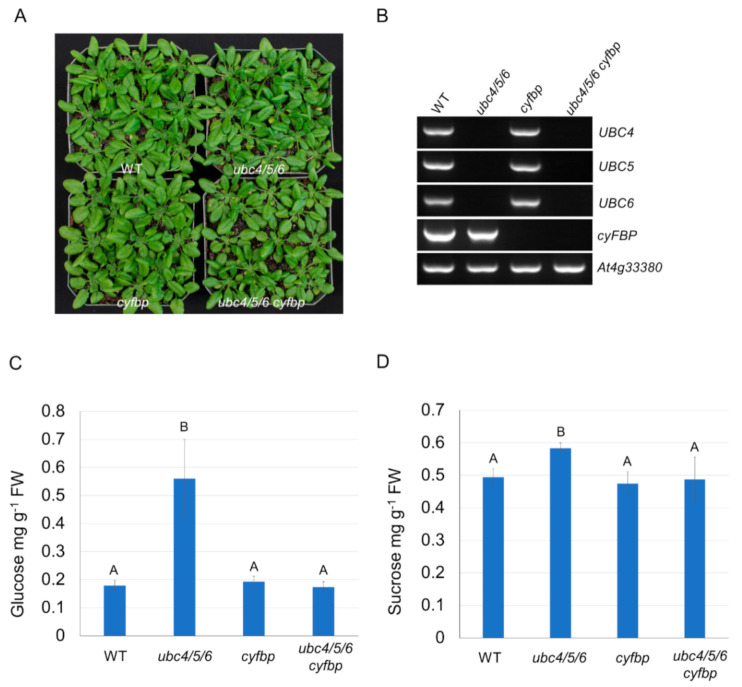
Plant growth and sugar analysis in the WT, *ubc4/5/6*, *cyfbp*, and *ubc4/5/6 cyfbp* mutant plants. (**A**) WT, *ubc4/5/6*, *cyfbp*, and *ubc4/5/6 cyfbp* mutant plants grown in pots and the picture was taken at the 5-week stage. (**B**) Genotyping of the *ubc4/5/6*, *cyfbp*, and *ubc4/5/6 cyfbp* mutant lines by genomic PCR with the WT as the control. The plants lines are indicated above the panels and specific genes analyzed are indicated at the right of the panels. The gene *At4g33380* was used as a control. (**C**) Quantitative analysis of the glucose level in the WT, *ubc4/5/6*, *cyfbp*, and *ubc4/5/6 cyfbp* mutant plants. About 300 mg of leaf tissues from 30-days-old plants were used for the analysis. Each datum (in mg of glucose per gram of fresh tissues) represents the average of four biological replicates (error bar = standard deviation). Data were analyzed using one-way ANOVA and post-hoc Tukey test, and significant differences are indicated by different letters (upper case) at *p <* 0.01 level. (**D**) Quantification of the sucrose level in the WT, *ubc4/5/6*, *cyfbp*, and *ubc4/5/6 cyfbp* mutant plants. About 300 mg of leaf tissues from 30-day-old plants were used for the analysis. Each datum (in mg of sucrose per gram of fresh tissues) represents the average of four biological replicates. The error bars indicate standard deviations. Data were analyzed using one-way ANOVA and post-hoc Tukey test, and significant differences are indicated by different letters (upper case) at *p <* 0.05 level.

Since the cytosolic FBPase is a key enzyme in sugar metabolism, and further down- or up-regulation of this enzyme or its homologs alters the levels of soluble sugars in different plant species [38], we asked whether the higher sugar levels observed in the *ubc4/5/6* mutant plants were due to an increased cyFBP level. Thus, we crossed the *ubc4/5/6* mutant with a *cyfbp* mutant in which *cyFBP* was inactivated [39], and obtained the quadruple *ubc4/5/6 cyfbp* mutant (Figure 5A,B). Quantitative analysis revealed that the glucose and sucrose levels in the quadruple mutant were similar to those in the WT plants, while the levels were significantly higher in the *ubc4/5/6* mutant (Figure 5C,D), suggesting that the increased cyFBP level was responsible for the increased levels of glucose and sucrose in the *ubc4/5/6* triple mutant.

### 2.5. UBC4/5/6 Play a Role in Leaf Senescence

**Figure 6:**
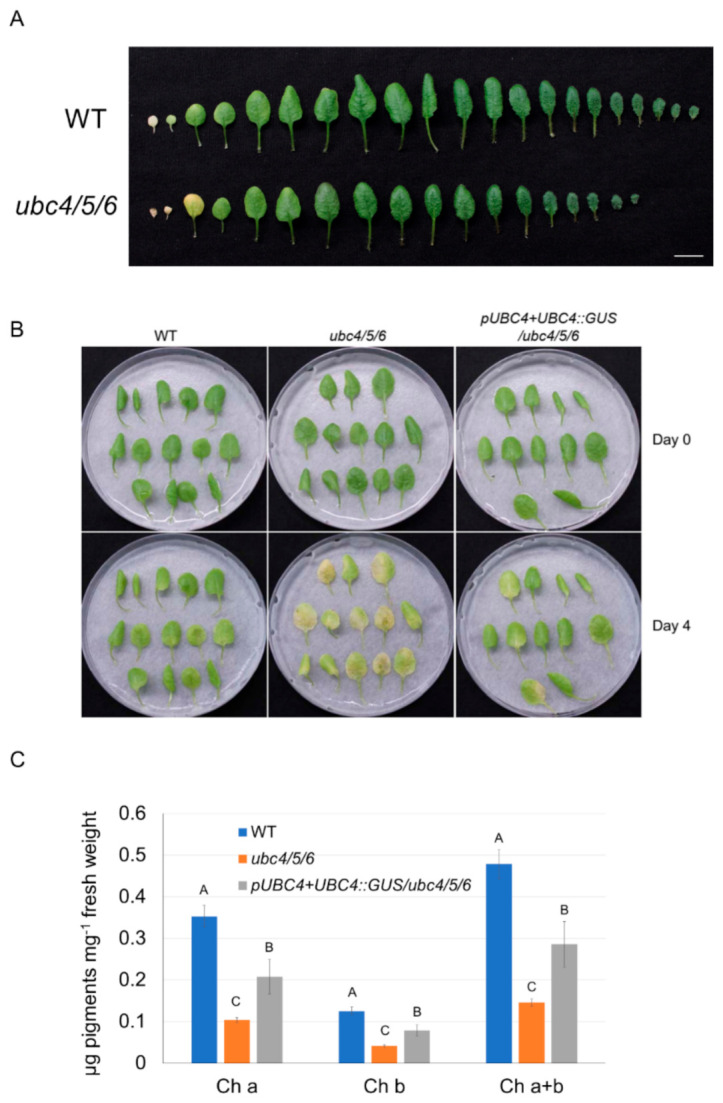
Analysis of early leaf senescence phenotype in the *ubc4/5/6* triple mutant plants. (**A**) Individual leaves were detached from representative WT and *ubc4/5/6* mutant plants at the 5-week stage and are shown from the oldest to the youngest. Scale bar: 1 cm. (**B**) Dark-induced senescence of detached leaves of the WT, *ubc4/5/6* mutant, and the *ubc4/5/6* mutant expressing *pUBC4::UBC4-GUS*. The second pair of true leaves were detached from 5-week-old plants and placed in Petri plates on a piece of filter paper with 4 mL of H_2_O added to keep the moisture. The plates were covered with a layer of lab bench absorbent paper to reduce the amount of light and incubated at room temperature for 4 days. Pictures were taken before (Day 0) and after the dark treatment (Day 4). (**C**) Chlorophyll content analysis of the detached leaves after the 4-day dark treatment in (**B**). Each datum (in μg of pigments per mg of fresh weight) represents the average of three replicate samples (with each sample having five detached leaves analyzed). The error bars indicate standard deviations. Data were analyzed using one-way ANOVA and post-hoc Tukey test, and significant differences are indicated by different letters (upper case) at *p <* 0.01 level.

**Figure 7:**
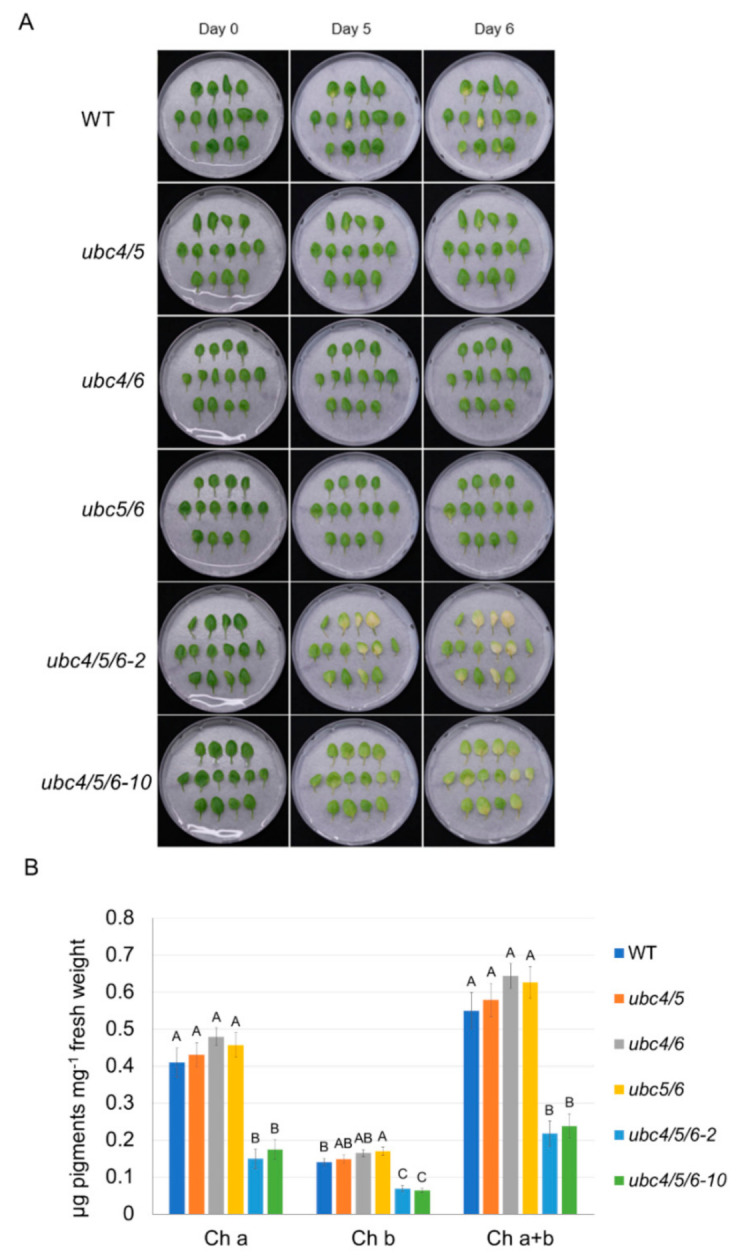
Analysis of early leaf senescence phenotype in different double and triple mutants of *UBC4*, *UBC5*, and *UBC6*. (**A**) Dark-induced senescence of detached leaves from 5-week plants of the WT, *ubc4/5*, *ubc4/6*, *ubc5/6* double mutants, and two lines of the *ubc4/5/*6 triple mutant (*ubc4/5/*6-2 and *ubc4/5/*6-10). The second pair of true leaves were detached from plants and used in the assay. They were placed in Petri plates on a piece of filter paper with 4 mL of H_2_O added. These plates were covered with a layer of lab bench absorbent paper and incubated at room temperature for 6 days. Pictures were taken before (Day 0) and after the dark treatment (Day 5 and Day 6). (**B**) Chlorophyll content analysis of the detached leaves after the 6-day dark treatment in (**A**). Each datum (in μg of pigments mg^−1^ fresh weight) represents the average of three replicate samples (with each sample having five detached leaves). The error bars indicate standard deviations. Data were analyzed using one-way ANOVA and post-hoc Tukey test, and significant differences are indicated by different letters (upper case) at *p <* 0.01 level.

Although leaves of the *ubc4/5/6* mutant plants appeared slightly greener than the WT plants before bolting, at the late stages of plant development the older leaves in particular appeared to senesce earlier compared to the WT plants. For instance, the first pair of mutant leaves showed greater senescence (Figure 6A). To further study this senescence-related phenotype, we analyzed detached leaves in a dark-induced senescence (DIS) assay. The second pair of leaves from 5-week-old plants were detached and incubated on a wet filter paper in Petri plates covered with a layer of lab bench absorbent paper to reduce the amount of light. After a 4-day dark incubation, the leaves of the *ubc4/5/6* mutant plants showed much greater senescence, with more yellow leaves and significantly lower chlorophyll contents (lower chlorophylls a and b) compared to the WT leaves (Figure 6B,C). The early senescence phenotype of the triple mutant was at least partially rescued by the expression of *pUBC4::UBC4-GUS* (Figure 6B,C). Furthermore, we also analyzed three different double mutant lines (*ubc4/5*, *ubc4/6*, and *ubc5/6*) which did not display the accelerated leaf senescence phenotype, as shown by the two triple mutant lines (*ubc4/5/6-2* and *ubc4/5/6-10*), indicating a functional redundancy among these E2s (Figure 7). Taken together, these results indicate an important role of UBC4/5/6 in leaf senescence during late plant development.

### 2.6. cyFBP Is Not Involved in the Accelerated Leaf Senescence Phenotype of the ubc4/5/6 Mutant

**Figure 8:**
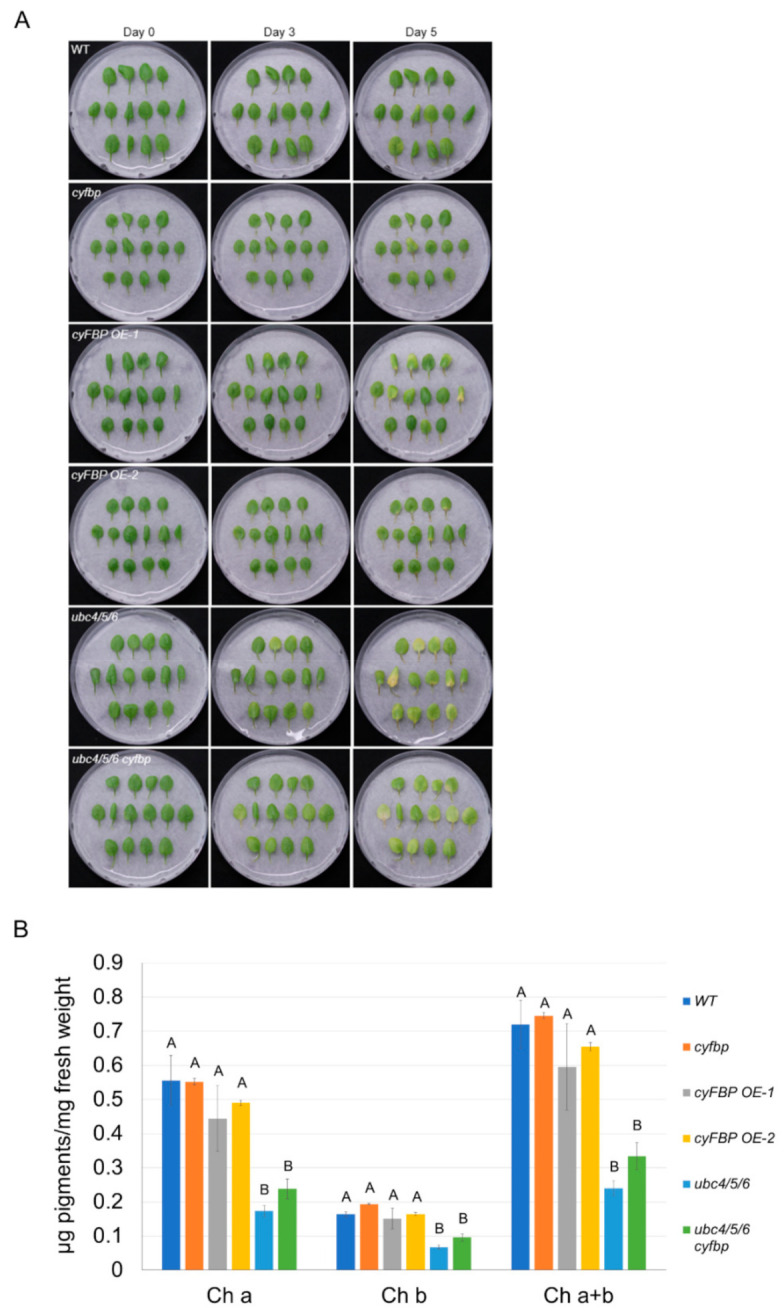
Knocking out *cyFBP* did not rescue the early leaf senescence phenotype of the *ubc4/5/6* mutant. (**A**) Dark-induced senescence of detached leaves from 5-week plants of the WT, *cyfbp*, two *cyFBP*-overexpressing lines, *ubc4/5/6*, and *ubc4/5/6 cyfbp*. The second pair of true leaves were detached from the plants and placed in Petri plates which had a piece of filter paper and 4 mL of H_2_O. These plates were covered with a layer of lab bench absorbent paper and incubated at room temperature for 5 days. Pictures were taken before (Day 0) and after the dark treatment (Day 3 and Day 5). (**B**) Chlorophyll content analysis of the detached leaves after the 5-day dark treatment in (**A**). Each datum (in μg of pigments mg^−1^ fresh weight) represents the average of three replicate samples (with each sample having five detached leaves). The error bars indicate standard deviations. Data were analyzed using one-way ANOVA and post-hoc Tukey test, and significant differences are indicated by different letters (upper case) at *p <* 0.01 level.

Since the increased cyFBP level was found to be responsible for the changes in the sugar levels in the *ubc4/5/6* mutant and sugar levels play critical roles in leaf senescence [51], we further asked whether the increased cyFBP level is responsible for the early or accelerated leaf senescence phenotype of the *ubc4/5/6* mutant. Thus, we used the *ubc4/5/6 cyfbp* quadruple mutant in the DIS assay along with the *ubc4/5/6* mutant, *cyfbp* single mutant, and two *cyFBP* overexpressing lines. Unlike the *ubc4/5/6* mutant plants, neither the *cyfbp* single mutant nor the two *cyFBP*-overexpressing transgenic lines showed the accelerated leaf senescence phenotype (Figure 8A). In contrast, the *ubc4/5/6 cyfbp* quadruple mutant still showed the accelerated leaf senescence phenotype as the *ubc4/5/6* mutant. Furthermore, after the dark treatment, the chlorophyll contents in the *ubc4/5/6 cyfbp* quadruple mutant were similar to those in the *ubc4/5/6* triple mutant (Figure 8B). These results together suggest that the increased cyFBP is not the cause for the early or accelerated leaf senescence phenotype of the *ubc4/5/6* triple mutant.

## 3. Discussion

### 3.1. UBC4/5/6 Have an Important Function in Sugar Metabolism

The existence of the phylogenetic conservation of the different E2 subfamilies among plant and non-plant species implies distinct functions for the different plant E2 subfamilies. However, less attention has been paid to the functional specificity of plant E2s compared to the E3 ligases [5,52]. For a more comprehensive understanding of the specific roles of various components of the ubiquitination machinery in plants, it is important to identify the functions of those E2s which are currently unknown. In this study, we focused on the subfamily IV represented by Arabidopsis UBC4/5/6. Our analysis revealed that the levels of glucose, sucrose, and starch increased in the triple mutant and such mutant phenotypes were rescued by expressing *pUBC4::UBC4-GUS* in the mutant background. These results clearly indicate that UBC4/5/6 have an important function in sugar metabolism. To our knowledge, no other Arabidopsis E2s have been shown to have a major function in sugar metabolism. In addition, we also identified a function of these E2s in leaf senescence. Furthermore, the *ubc4/5/6* mutant plants appeared to have stronger freezing tolerance compared to the WT plants when they were treated at −10 °C for 1 h, although there was no difference between the triple mutant and WT plants when treated at −12 and −14 °C. The stronger freezing tolerance exhibited by the mutants plants at −10 °C was possibly due to the higher soluble sugar levels, since sugar metabolism is closely linked to cold tolerance in plants [49]. The *cyFBP* expression was induced by the cold treatment in Arabidopsis and plants with higher soluble sugars had an improved freezing tolerance [53].

### 3.2. UBC4/5/6 Are Involved in Regulating the Level of cyFBP

Currently, little is known about the regulation of important enzymes in plant sugar metabolism (either in glycolysis or gluconeogenesis) through protein ubiquitination. In yeast, the protein levels of three important gluconeogenic enzymes malate dehydrogenase (Mdh2), phosphoenolpyruvate carboxykinase (Pck1), and fructose-1,6-bisphosphatase (Fbp1) are regulated through ubiquitination by an E3 ligase complex called the Gid complex [54]. These gluconeogenic enzymes can be ubiquitinated and degraded when glucose is resupplied to the cells grown on a non-fermentable carbon source [54]. It is worth noting that yeast Ubc8 has been identified as a subunit of the Gid complex [34], and is evolutionarily related to Arabidopsis UBC4/5/6 (Figures S1 and S2), suggesting that these plant E2s may have a conserved function with the yeast Ubc8.

**Figure 9:**
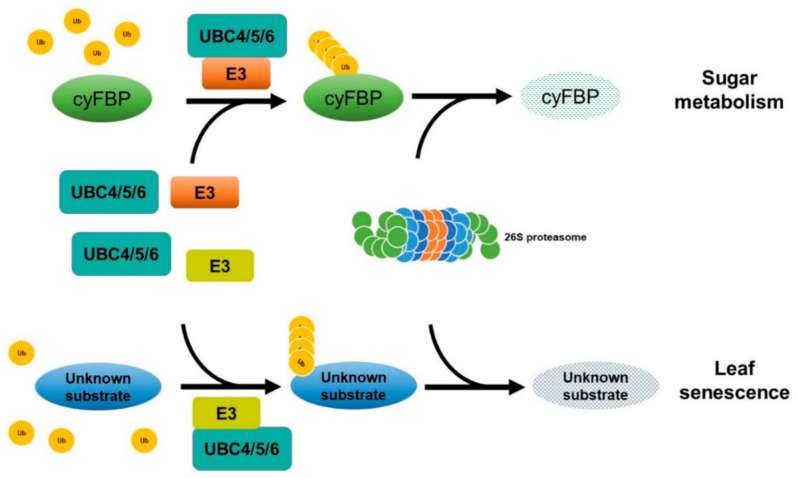
A model to show the functions of UBC4, UBC5, and UBC6 in sugar metabolism and leaf senescence. UBC4, UBC5, and UBC6, the three members of the E2 subfamily IV, together with an E3 yet to be identified, target and most likely ubiquitinate a key gluconeogenic enzyme, the cytosolic fructose-1, 6-bisphastase (cyFBP), which is involved in sugar metabolism in plants. When the three genes are inactivated, cyFBP accumulates, resulting in increased levels of glucose and sucrose in the leaves. Additionally, these three E2s also function to affect leaf senescence at late stages of plant development independent of cyFBP. The substrate and E3 involved remain to be uncovered.

Despite the importance of the cytoplasmic FBPases in sugar metabolism, little is known regarding how their protein levels are regulated in plants. The results from several large-scale studies to screen for ubiquitinated proteins in leaf tissues identified several enzymes involved in photosynthesis and carbohydrate metabolism, including the Arabidopsis cyFBP and related proteins in several other plant species [55,56,57,58], suggesting that they may be subjected to ubiquitination. However, the results from those large-scale studies on the possible ubiquitination of FBPases were not validated by further analysis and more importantly the functional significance is unknown. In our study, we found that the ubc4/5/6 mutant plants had a high level of cyFBP, suggesting that cyFBP level is regulated by the ubiquitination machinery in vivo. The finding that the degradation of recombinant His-cyFBP in a cell-free degradation assay was inhibited by the proteasome inhibitor MG132 indicated that the degradation of His-cyFBP was through the 26S proteasome. Conversely, adding ATP to the total protein extract promoted the degradation of cyFBP, consistent with the requirement of ATP during protein ubiquitination. Furthermore, proteins larger than the HA-cyFBP band could be pulled down by HA-conjugated beads from the total protein extract of the transgenic plants overexpressing *HA-cyFBP*, suggesting that HA-cyFBP protein is subjected to ubiquitination in vivo. These different lines of evidence demonstrate that cyFBP protein level is regulated through ubiquitination, most likely mediated by UBC4/5/6 (Figure 9).

In Arabidopsis, the inactivation of either *cyFBP* or *cFBP1* or both resulted in a higher F1,6BP level since the catalytic conversion of F1,6BP to F6P was affected. In the *cyfbp* single mutant, the sucrose level was only affected at some time points during the day [39]. It has been suggested that reduced sucrose synthesis in the cytosol of the *cyfbp* mutant is compensated by more export of hexoses or hexose-phosphates from the chloroplasts as a result of *cFBP1* up-regulation and increased starch turnover [39]. Thus, the growth of the *cyfbp* mutant was similar to the WT. However, the specific mechanism for the compensation effect between *cyFBP* and *cFBP1* is not clear. Nevertheless, two studies of transgenic overexpression have reported that increased FBPase expression in the cytosol could increase sucrose synthesis and enhance plant growth [41,42], which is consistent with our finding that the Arabidopsis *ubc4/5/6* mutant plants had an increased level of cyFBP protein and higher soluble sugar levels (glucose, fructose, and sucrose). Furthermore, our finding that the soluble sugar levels in the *ubc4/5/6* mutant returned to normal levels when *cyFBP* was inactivated strongly suggests that the increased soluble sugar levels in the *ubc4/5/6* mutant were due to an increased cyFBP level. These results indicate that UBC4/5/6 function in sugar metabolism through regulating the level of cyFBP, thus unravelling a novel mechanism for cyFBP regulation in plants.

Interestingly, the level of cFBP1 was not affected in the *ubc4/5/6* triple mutant. Furthermore, adding ATP did not promote the degradation of endogenous cFBP1 in the cell-free degradation assay. These findings suggest that UBC4/5/6 are not involved in the ubiquitination and regulation of cFBP1 in the chloroplast. The reason for the three E2s to be involved in regulating the level of cyFBP, but not that of cFBP1, could either be due to the lack of subcellular localization of these E2s in the chloroplast or lack of an E3 ligase in the chloroplast that confers the substrate specificity or to both possibilities. There is some evidence for the first possibility. First, no chloroplast import signal sequences are found in these E2 protein sequences. Second, the YFP signal of the transgenic line (*pUBC4::UBC4-YFP*) was observed in the cytosol and nucleus in leaves, but not in the chloroplast (Figure S8). Furthermore, consistent with these results, several large-scale studies on the ubiquitinated proteins in leaves only identified the cytosolic FBPases in Arabidopsis and other species, but not the chloroplastic FBPases [55,56,57,58]. These results suggest that the cytoplasmic and chloroplastic FBPases are regulated differently in plants.

### 3.3. UBC4/5/6 also Function in Leaf Senescence

Leaf senescence is a programed biological process at the late stages of leaf development and is associated with protein degradation. Ubiquitination-mediated protein degradation is one of the main pathways to regulate the removal of organelles and abnormal proteins during senescence. E3 ligases have been reported as key regulators during leaf senescence [59]. For example, the HECT-type E3 ligase, UPL5 (UBIQUITIN PROTEIN LIGASE 5) can ubiquitinate and degrade an important transcription factor WRKY53 during senescence. The *upl5* mutants showed an early leaf senescence phenotype with an increased WRKY53 protein level [60]. Regarding the specific E2s, only UBC24 has been implicated in leaf senescence. It has been reported that UBC24 is the cognate E2 for the E3 ligase NLA (NITROGEN LIMITATION ADAPTION) and the E2-E3 module UBC24-NLA mediates the ubiquitination and degradation of a key senescence regulator ORE1 (ORESARA 1) under nitrogen deficiency [23]. In this study, we observed that leaves of the *ubc4/5/6* mutant plants showed early leaf senescence and the detached mutant leaves showed accelerated senescence compared to the WT plants, while the three different double mutants did not show the accelerated leaf senescence phenotype in the DIS. Further, the expression of UBC4-GUS could partially rescue the mutant phenotype. In addition, the three different double mutants did not show the accelerated leaf senescence phenotype as the triple mutant in the DIS, suggesting functional redundancy among UBC4/5/6. These results thus have identified another interesting function of the E2s in addition to their function in sugar metabolism.

Our finding that the function of UBC4/5/6 in sugar metabolism is mainly through regulating the level of cyFBP raises an interesting question whether the accelerated leaf senescence phenotype of the *ubc4/5/6* mutant is also through cyFBP. The following lines of evidence, however, suggest that the early leaf senescence phenotype in the *ubc4/5/6* triple mutant is cyFBP-independent. First, transgenic plants overexpressing *cyFBP* did not display an early leaf senescence phenotype under the dark treatment compared to the WT plants. Second, the *cyfbp* mutant did not show the accelerated leaf senescence in the DIS assay. Third, the *ubc4/5/6 cyfbp* quadruple mutant still showed the accelerated leaf senescence phenotype as the *ubc4/5/6* triple mutant while the sugar levels in the quadruple mutant were similar to the levels in the WT plants. Therefore, we propose that UBC4/5/6 have two lines of different biological functions, in sugar metabolism through cyFBP and in leaf senescence through a cyFBP-independent mechanism (Figure 9). The function in regulating cyFBP is conserved with that of yeast Ubc8 while the function in leaf senescence may represent a more distinct function of these E2s in plants. Thus, the present results have advanced our understanding of this subfamily of plant E2s by discovering the functions of the three Arabidopsis E2s and suggesting similar functions of related E2s in other plants.

## 4. Materials and Methods

### 4.1. Plant Materials and Growth Conditions

*Arabidopsis thaliana* ecotype “Columbia-0” and its mutant lines were grown in a growth chamber (20 °C constant, 12/12 h day/night photoperiod with a fluence rate of 90 ± 10 μmol m^−2^ s^−1^). Arabidopsis T-DNA insertion lines used in this study were obtained from the Arabidopsis Biological Resources Center (ABRC), including *ubc4-1* (SALK_085489), *ubc5-1* (SALK_051162C), *ubc6-1* (SALK_026155C), *cyfbp* (SALK_064456), and *cfbp1-2* (WiscDsLoxHs084_10B). The higher order mutants were obtained by crossing between different single, double, or triple mutants. Homozygous lines were obtained and used in further analyses.

### 4.2. Isolation and Analysis of Plant Genomic DNA and RNA

Genomic DNA samples were isolated from leaf tissues as described [61].

For total RNA isolation, leaf tissues of 5-week-old plants or 2-week-old seedlings were collected and ground in liquid nitrogen. RNA samples were isolated from about 100 mg of tissues using Trizol reagent (Thermo Fisher Scientific, Ottawa, ON, Canada) following the manufacturer’s instructions and the RNA concentration was determined with a NanoDrop 2000 Spectrophotometer (Thermo Fisher Scientific). cDNAs were synthesized using the ThermoScript RT-PCR system (Thermo Fisher Scientific, formerly Invitrogen) following the manufacturer’s instructions. Gene-specific primers were used to determine the presence of the genomic fragment or the transcript of a gene in DNA or cDNA samples by PCR, respectively. The gene *At4g33380* was used as an internal control [29]. The primers used are listed in Table S1.

### 4.3. Iodine Staining of Starch

Arabidopsis plants at the 5-week stage were collected at the end of the day and bleached in boiling ethanol (80% *v*/*v*) for 3 min and this step was repeated two more times. The plants were then washed with ddH_2_O and stained with a Lugol’s iodine solution (5% iodine *w*/*v* and 10% potassium iodide *w*/*v*) for 2 min. After that, the plants were briefly washed with ddH_2_O before observation.

### 4.4. Quantitative Analysis of Soluble Sugar and Starch Levels

Soluble sugars were extracted as described [62,63] with some modifications. Briefly, about 300 mg of leaf tissues of 3- to 4-week-old plants were collected at the end of the day and ground to fine powder in liquid nitrogen, and then extracted in 3 mL of 80% ethanol by incubating at 95 °C for 10 min in a water bath. Samples were allowed to cool down before centrifugation for 10 min at 5000× *g* to collect the supernatant. This step was repeated two more times. The supernatant from the three rounds of extraction were combined for soluble sugar analysis and the pellet was saved for starch analysis. Double distilled water and chloroform were added into the supernatant (supernatant:H_2_O:chloroform = 4:2:1) to remove pigments including chlorophylls. The samples were then centrifuged for 10 min at 5000× *g* and the aqueous phase containing soluble sugars was transferred to a new centrifuge tube for further analysis. Fructose, glucose, and sucrose levels were determined with a Sucrose, D-fructose, and D-glucose assay kit (Megazyme, Wicklow, Ireland) following the manufacturer’s instructions. For starch extraction, five ml of ddH_2_O was added to suspend the pellet and then the samples were incubated in a 95 °C water bath for 30 min. From the homogenate, 0.5 mL was removed and mixed with 0.5 mL of sodium acetate (pH 5.5) containing 5 U α-amylase and 10 U α-amyloglucosidase. The reaction solution was incubated at 37 °C for 24 h to convert starch to glucose, which was determined using the Sucrose, D-fructose, and D-glucose assay kit (Megazyme).

### 4.5. Freezing Tolerance

Freezing tolerance was determined as described [64] with some modifications. Four-week-old plants grown at 20 °C were shifted to a controlled growth chamber at 4 °C for 7 days with a 12 h photoperiod and then three pots per test temperature were transferred to a programmable freezer (Cincinnati Sub-Zero, Sharonville, OH, USA) set at 0 °C for 30 min and then cooled at a rate of 2 °C/h to −4 °C. Plants were misted with an ice nucleation active substance and held at −4 °C for 2 h. The programmable freezer was cooled 2 °C/h to −10, −12, or −14 °C. Plants were treated for 1 h at the treatment temperature and then transferred to a dark room at 4 °C for 24 h before being transferred to a 20 °C chamber with a 12 h photoperiod. After 14 days, plant survival was scored as the proportion of plants to regrow new leaves from the meristem. Determinations were repeated for each genotype and treatment combination.

### 4.6. Dark-Induced Senescence (DIS) Assay and Chlorophyll Content Measurements

The dark-induced senescence (DIS) assay was performed as described [65] with modifications. The second pair of rosette leaves from 5-week-old plants were excised and placed in Petri dishes on a piece of filter paper with 4 mL of ddH_2_O added to keep the moisture. These plates were covered with a layer of lab bench absorbent paper (Versi-Dry, Thermo Scientific) to reduce the amount of light and were incubated at room temperature (21 ℃) for 4–6 days. Pictures were taken before and at different time points of the treatment.

Chlorophyll contents were quantified following a modified protocol [66]. The dark-treated leaves were incubated in DMSO for 2 h in a 60 °C water bath. Extracts were centrifuged at 12,000× *g* for 5 min at 4 °C and the supernatant was saved for the chlorophyll content analysis by measuring absorbance at 645 and 663 nm. The concentrations of chlorophyll a, chlorophyll b, and total chlorophyll were calculated using the following equation: chlorophyll a: 12.7 × A^663^ − 2.69 × A^645^; chlorophyll b: 22.9 × A^645^ − 4.68 × A^663^; total chlorophyll: 20.2 × A^645^ + 8.02 × A^663^.

### 4.7. Construct Preparation and Plant Transformation

For UBC4 protein expression in Arabidopsis, the *UBC4* genomic fragment (−1155 position upstream of ATG to +1661 position downstream from ATG) was amplified from WT genomic DNA and then cloned into a plant expression vector modified from *pCambia1300* with a GUS reporter [67], resulting in *pUBC4::UBC4-GUS*. For determining the subcellular expression of UBC4, a *YFP* fragment was cloned into *pUBC4::UBC4-GUS* to replace the *GUS* reporter gene, resulting in *pUBC4::UBC4-YFP*. For cyFBP protein expression in Arabidopsis, the full-length coding sequence of *cyFBP* amplified with primers 1411 and 1412 was cloned into the plant expression vector modified from pBI121 (Clontech) that has an HA (influenza hemaglutinin) tag behind the 35S promoter [68], resulting in *p35S::HA-cyFBP*. These constructs were introduced into WT or *ubc4/5/6* mutant plants and homozygous progeny plants were used in further analyses.

### 4.8. Plant Protein Extraction and Western Blotting

Proteins were extracted from Arabidopsis leaves and analyzed by Western blotting as described previously [68]. Briefly, leaf samples or seedlings were ground and suspended in the extraction buffer (50 mM Tris-HCl pH 8.0, 200 mM NaCl, 10 mM DTT (dithiothreitol), protease inhibitor cocktail (MilliporeSigma, Oakville, Canada, #P9599)). Samples were centrifuged twice at 14,000× *g* at 4 °C for 5 min to obtain the supernatant. The total protein concentration was determined [69] before further analysis.

The protein samples were subjected to electrophoresis in 10% SDS-PAGE (sodium dodecyl sulphate-polyacrylamide gel electrophoresis) gels run at 100 V for 2 h, before being electroblotted onto a PVDF membrane (Bio-Rad, Mississauga, Canada) at 80 V for 4 h at 4 °C. The PVDF membrane blots were incubated sequentially with a blocking buffer (5% (*w*/*v*) skim milk in 1X PBS buffer) for 1 h, with the desired primary antibodies overnight, and then with the anti-IgG-HRP (horseradish peroxidase) conjugate for 1 h. The primary antibodies used were: anti-cyFBP (1: 10,000; Agrisera, Vännäs, Sweden, AS04 043), anti-cFBP1 (1: 10,000; Agrisera, AS19 4319), anti-HA (1: 7000; Santa Cruz, sc-7392), anti-β-actin (1: 10,000; Sigma, A5316), anti-Ub (1: 10,000; Cell Signaling Technology, Danvers, USA, P4D1), and anti-His (1: 10,000; Thermo Fisher Scientific, MA1-21315). The secondary antibodies used were an HRP-conjugated goat anti-mouse IgG antibody (Bio-Rad) and an HRP-conjugated goat anti-rabbit IgG antibody (Abcam, Toronto, Canada). Signals were visualized using the ECL Prime reagent (Cytiva, Mississauga, Canada) following the manufacturer’s instructions. For quantitative analysis, the intensity of each band on membrane blots was measured using ImageJ software 1.49v and calculated with the average of the control bands set at 1.0.

### 4.9. Cell-Free Protein Degradation Assay

The cell-free protein degradation assay was performed as described with some modifications [50]. His-cyFBP recombinant protein was expressed and purified from *Escherichia coli* strain BL21 (DE3) (Stratagene, San Diego, USA) using Ni-NTA spin columns (Qiagen, Toronto, Canada) following the manufacturer’s instructions. For proteasome inhibitor treatments, about 300 ng of His-cyFBP protein was incubated in 100 μL of solution containing 500 μg of total proteins with or without 100 μM MG132 at room temperature. Samples were collected at the indicated time points and the amount of His-cyFBP was determined by Western blotting using an anti-His antibody as described above. For the degradation assay with the addition of ATP, the total protein extracts were prepared from 2-week-old seedlings of the transgenic *p35S::HA-cyFBP* plants. About 60 μg of total proteins were incubated at room temperature with or without 5 mM ATP for the indicated lengths of time. The samples were added with a 4X SDS loading buffer to stop the reaction and were then analyzed by Western blotting using anti-cyFBP, anti-cFBP1, and anti-HA antibodies.

### 4.10. HA-cyFBP Protein Pulldown

The HA-cyFBP protein was pulled down from the total protein extract as described [70]. The anti-HA agarose beads (Sigma-Aldrich) were added into the total protein extract (1 mg of total proteins) and incubated at 4 °C for 1 h (on a tumbler). The beads were then washed twice with the wash buffer (10 mM Tris-HCl at pH 8.0, 5 mM NaCl, 0.05% Tween, 0.5 mM dithiothreitol, and 0.5 mg ml^−1^ BSA). The bound proteins were eluted with 1 mg ml^−1^ HA peptide (GenScript, Piscataway, USA). After adding the 4X SDS loading buffer and heat treatment at 95 °C for 5 min, the samples were then analyzed by electrophoresis and Western blotting for HA-cyFBP and ubiquitinated HA-cyFBP as described [70].

## Data Availability

All data have been provided in the manuscript as main figures and tables or as Supplementary Materials.

## Supplementary Materials

The following supporting information can be downloaded at: https://www.mdpi.com/article/10.3390/ijms231911143/s1.
