# Supplementary material for: Arabidopsis Ubiquitin-Conjugating Enzymes UBC4, UBC5, and UBC6 Have Major Functions in Sugar Metabolism and Leaf Senescence
Source: Int J Mol Sci. 2022 Sep 22;23(19):11143. doi: 10.3390/ijms231911143 (PMC9569852; doi:10.3390/ijms231911143)
Supplement: Supplementary file 1 [file ijms-23-11143-s001.zip › ijms-1915073-supplementary.pdf]

Supplementary Materials

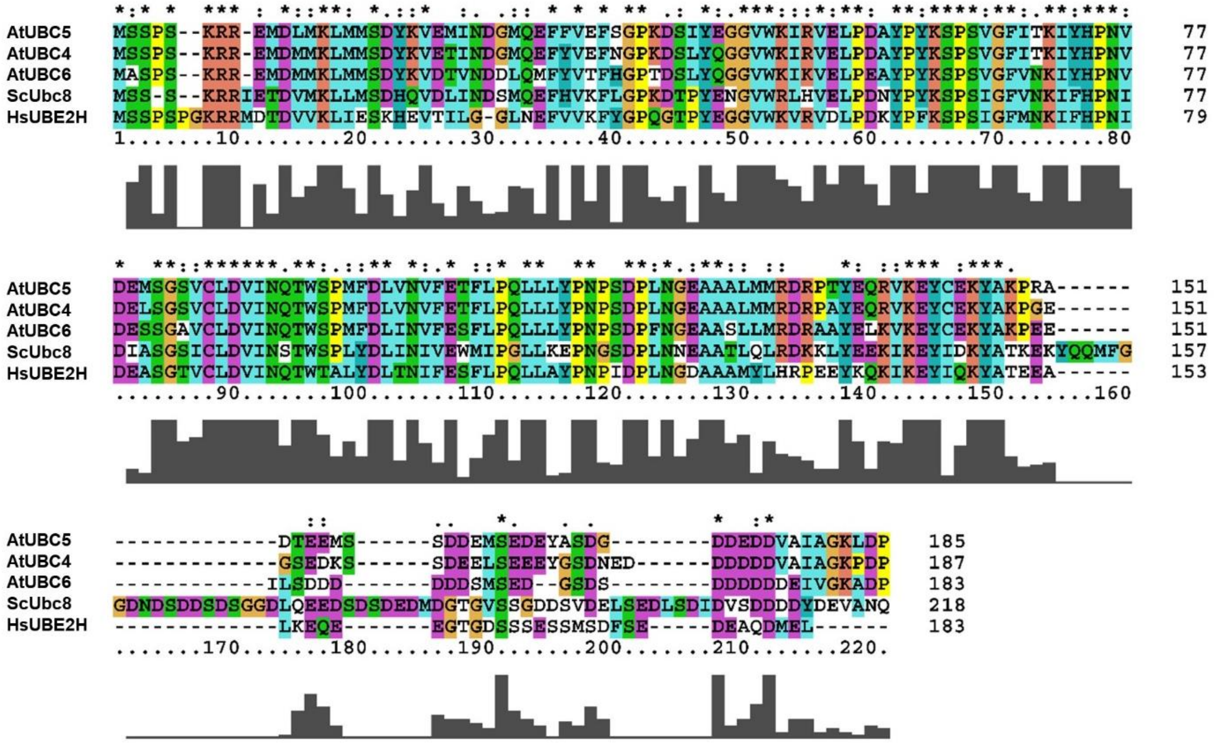

Figure S1 Protein sequence alignment of Arabidopsis UBC4, UBC5 and UBC6.
Arabidopsis UBC4, UBC5 and UBC6, and the homologs from baker yeast (ScUbc8) and human (HsUBE2H) are included in the analysis. The alignment was created using ClustalX. The sequences used are: AtUBC4 (P42748); AtUBC5 (P42749); AtUBC6 (P42750); ScUbc8 (P28263); HsUBE2H (P62256).

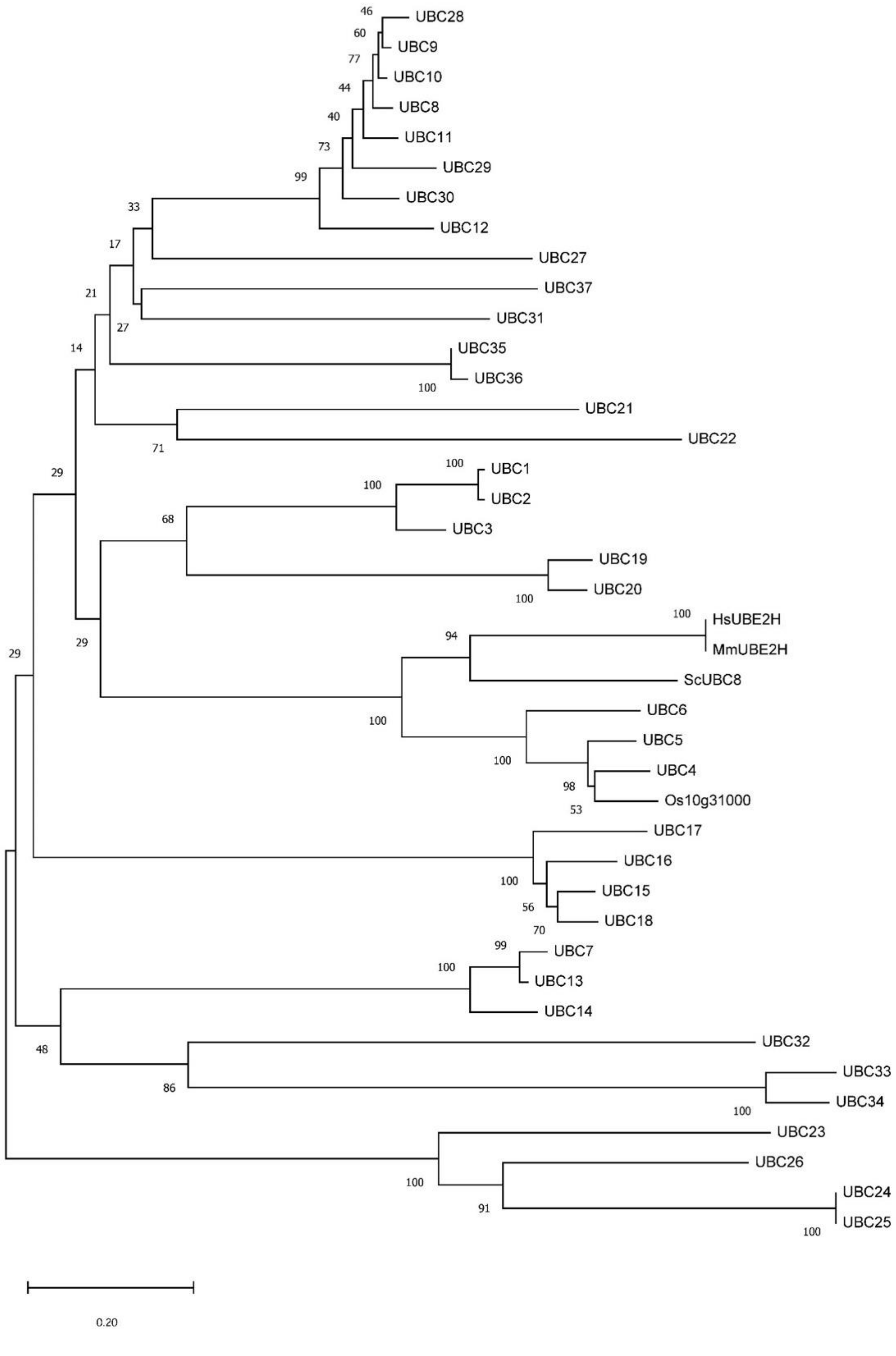

Figure S2 Phylogenetic analysis of Arabidopsis ubiquitin-conjugating enzymes (E2s) with several E2s from other species related to UBC4, UBC5 and UBC6.

The 37 Arabidopsis E2s and the homologs of Arabidopsis UBC4/5/6 from several plant and non-plant species were included (HsUBE2H from human, MmUBE2H from mouse, ScUbc8 from baker yeast, and Os10g31000 from rice). The analysis was performed using ClustalX and the phylogenetic tree was created using MAGA. Bootstrap values from 500 replications for each branch are shown.

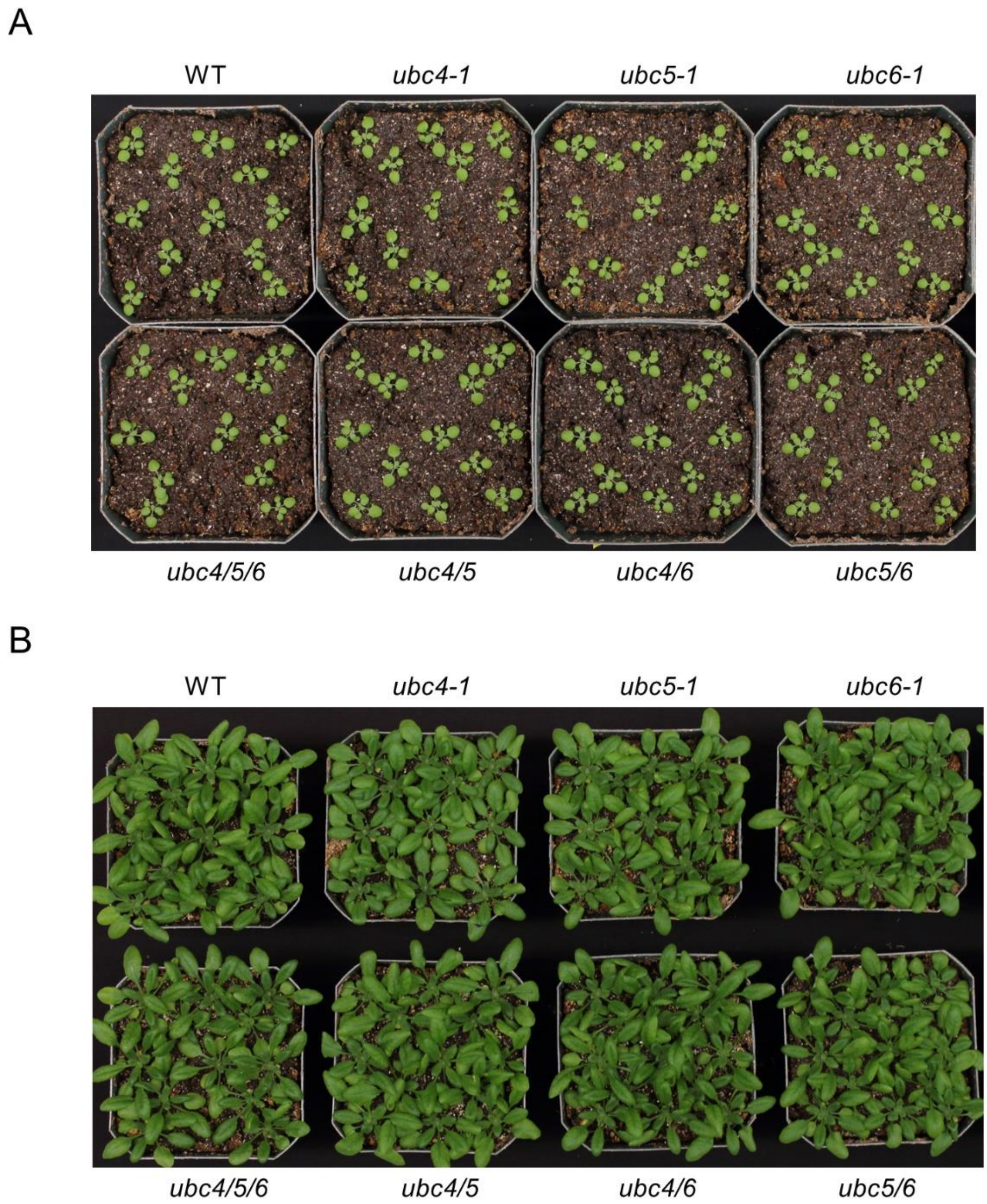

Figure S3 Images of the WT, *ubc4-1*, *ubc5-1*, *ubc6-1, ubc4/5, ubc4/6, ubc5/6,* and *ubc4/5/6* mutant plants grown in pots at the 2-week (A) and 30-day (B) stages.

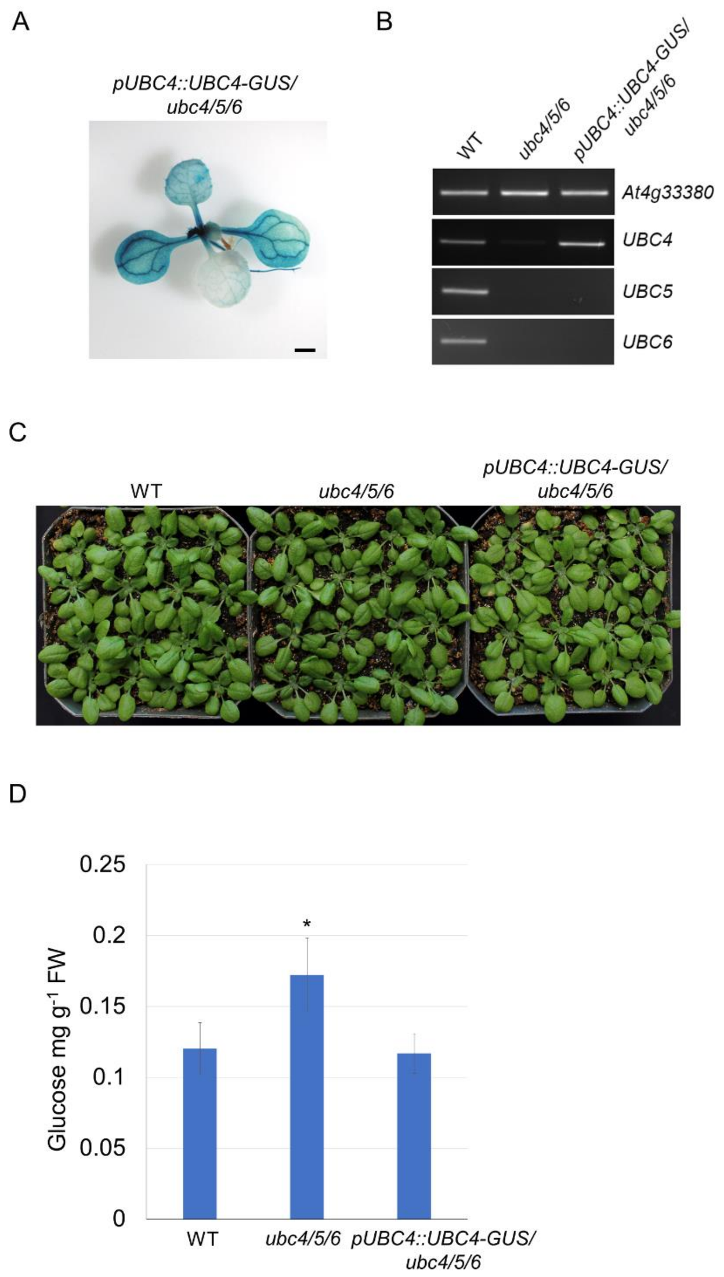

Figure S4 Complementation of the *ubc4/5/6* triple mutant by *pUBC4::UBC4-GUS*.
(A) GUS staining of an *ubc4/5/6* mutant seedling expressing *pUBC4::UBC4-GUS.* Scale bar: 1 mm. (B) Analysis of *UBC4, UBC5* and *UBC6* transcripts by RT-PCR in the *ubc4/5/6* mutant and *ubc4/5/6* mutant expressing *pUBC4::UBC4-GUS*. Gene-specific primers were used to detect the transcripts of each gene, and the transcript level of *At4g33380* was used as a reference. (C) Plants of the WT, *ubc4/5/6* mutant and *ubc4/5/6* mutant expressing *pUBC4::UBC4-GUS.* Sixteen plants were grown in each pot and the image was taken at the 30-day stage. (D) Analysis of the glucose level in the WT, *ubc4/5/6* mutant and *ubc4/5/6* mutant expressing *pUBC4::UBC4-GUS*. About 300 mg leaf tissues from 4-week-old plants were used for the glucose analysis. Each datum presents the average of four biological replicates. The error bars indicate standard deviations. Student's *t*-test was performed to determine whether there is a significant difference. *: P-value <0.05.

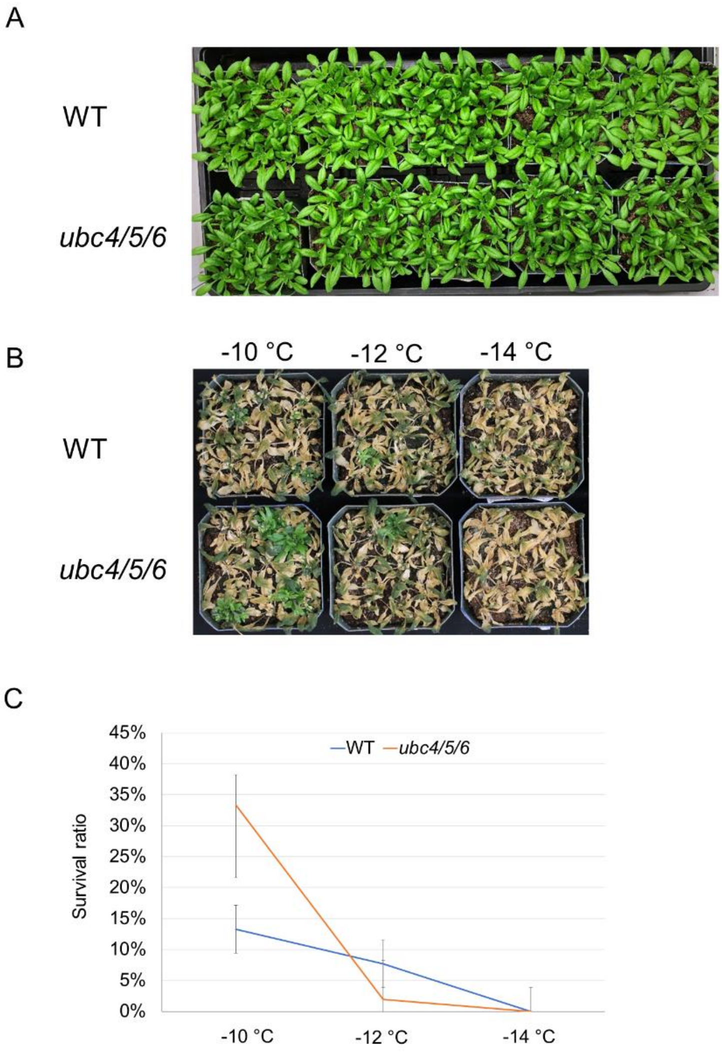

Figure S5 Analysis of freezing tolerance in the WT and *ubc4/5/6* mutant.
(A) Four-week-old WT and *ubc4/5/6* mutant plants before the freezing treatment. (B) Representative images showing the plant survival of the WT and *ubc4/5/6* mutant after the freezing treatment. The four-week-old Arabidopsis plants grown at 20 °C were shifted to 4 °C for 7 days and then transferred to a programmable freezer for the freezing treatment at the indicated temperatures for 1h. The number of plants survived was surveyed after 14 days and a plant was considered to have survived if there were new leaves re-growing from the meristem. (C) Quantitative data showing the percentage of survival of the WT and *ubc4/5/6* mutant plants after the freezing treatment. The error bars indicate standard deviations.

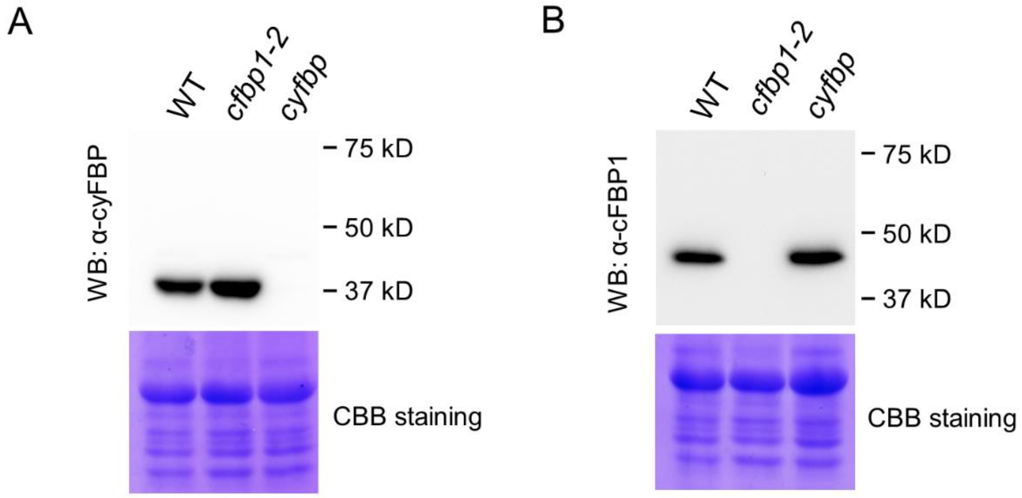

Figure S6 Specificity analysis of the antibodies against cyFBP and cFBP1.
Total protein extracts from the WT, *cyfbp1* and *cfbp1-2* mutant plants were used in Western blotting to test the specificity of the antibodies raised against cyFBP (A) and cFBP1 (B). The blots were blotted with the primary antibodies against cyFBP and cFBP1 (both from Agrisera) respectively, a secondary HRP-conjugated goat anti-rabbit IgG antibody (Abcam) and visualized with the ECL Prime reagent (Cytiva). The plant lines used are indicated above the panels. The molecular markers are indicated at the right. The Coomassie Brilliant Blue (CBB) stained gels are presented (the lower panel) to show the evenness of sample loading.

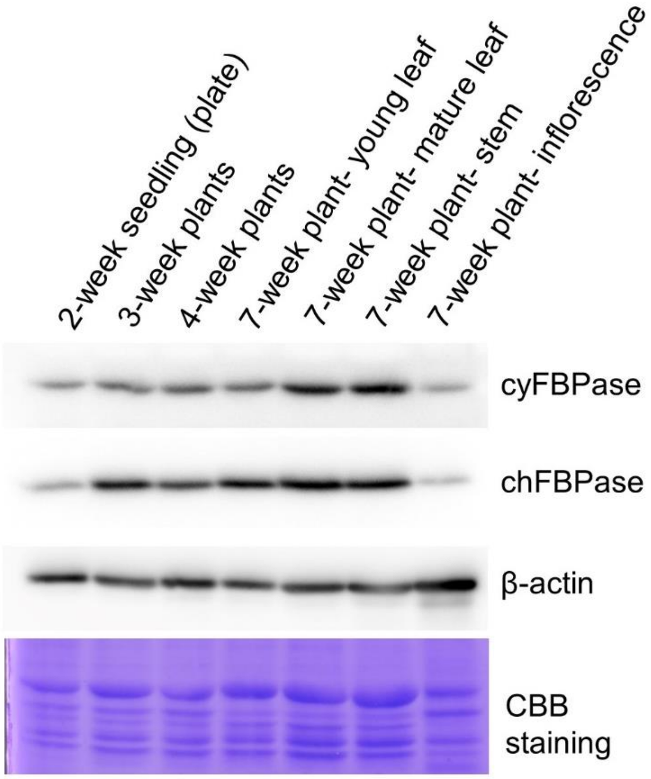

Figure S7 cyFBP and cFBP1 protein levels in Arabidopsis tissues at different developmental stages.

Total protein extracts were prepared from the indicated tissues at different developmental stages of the WT plants and analyzed using Western blotting with the antibodies against cyFBP and cFBP1. The different samples are indicated above the panel. β-actin was analyzed as a loading reference. Coomassie Brilliant Blue (CBB) staining of the gel at the lower panel was also used to show the evenness of sample loading.

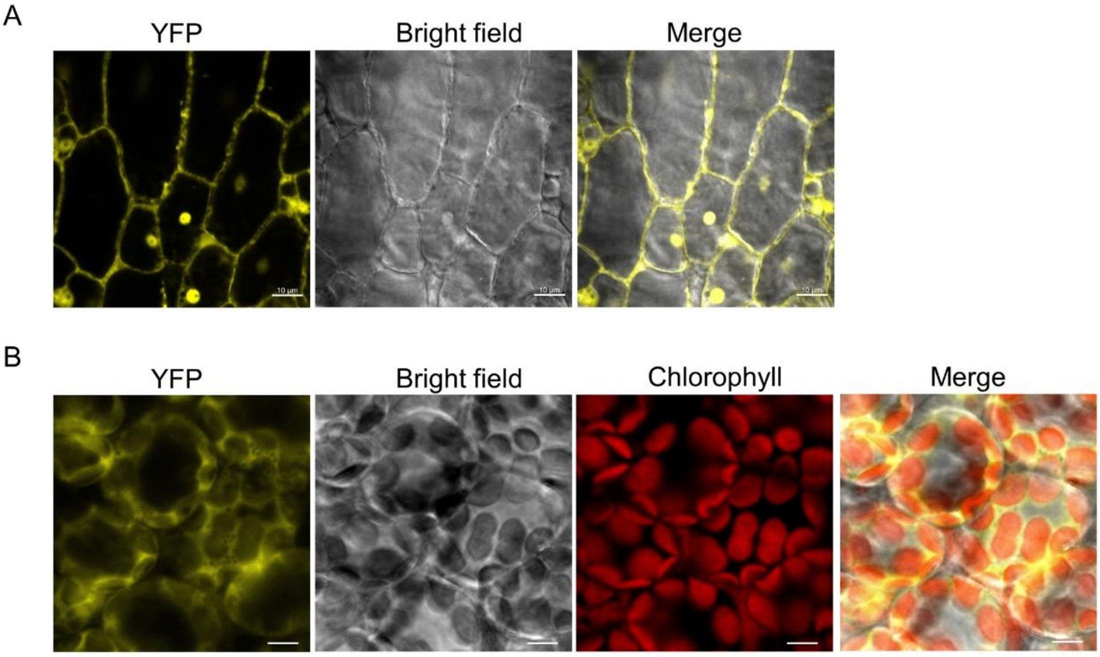

Figure S8 Subcellular localization of UBC4.

The seeds of *pUBC4::UBC4-YFP* transgenic line were sterilized and plated on the 1/2 Murashige and Skoog (MS) plates with 0.7% agar. After 2 weeks, the expression of UBC4-YFP in epidermal cells (A) and mesophyll cells (B) were analyzed in the leaf tissue under a confocal laser scanning microscope (ZEISS LSM880). The excitation/emission wavelengths were set as 514/520-580 for YFP and 488/670-680 for chlorophylls. Different channels are indicated at the top of the images. Scale bars: (A) 10 μm and (B) 5 μm.

Table S1. Sequences of primers used for genotyping mutants, RT-PCR and cloning

Description	Primer name	Sequences
At5g41350 (UBC4)	1451 (F)	CAG TGT CGA CAA TGT CTT CGC CAA GCA AAC
	1452 (R)	CAG TGC GGC CGC TCA AGG ATC TGG TTT GCC A
At1g63800 (UBC5)	1453 (F)	CAG TGT CGA CAA TGT CTT CGC CGA GCA AG
	1396 (R)	CAT GCG GCC GCT TAA GGA TCA AGT TTA CCG GC
At2g46040 (UBC6)	1454 (F)	CAG TGT CGA CAA TGG CGT CGC CGA GCA AA
	1455 (R)	CAG TGC GGC CGC TCA TGG ATC GGC TTT CCC A
At4g33380	471 (F)	ATG AGA AGC TGG AGG AAG C
	472 (R)	TCA AGC CGT TAC AAC ACC
At5g41350 (UBC4) promoter and coding region	1441 (F)	CAT CTG CAG TCG AAA CTT GGT TTG TTT AGT
	1442 (R)	CAG TCT CGA GGC AGG ATC TGG TTT GCC AGC
At1g43670 (cyFBP)	1411 (F)	CAG TGT CGA CAA TGG ATC ACG CAG CAG ATG
	1412 (R)	CAG TGC GGC CGC TTA GTT CTT CTT TTC CTC CTC
